# Comparative Transcriptomics and Metabolomics Analysis Revealed the Mechanism of Exogenous Salicylic Acid Improving the Cold Tolerance of Walnut

**DOI:** 10.3390/ijms27072948

**Published:** 2026-03-24

**Authors:** Jingmiao Li, Wenhao He, Feng Liu, Youchao He, Jianxun Qi, Baojun Zhao, Yunqi Zhang, Shuchai Su

**Affiliations:** 1 College of Forestry, Beijing Forestry University, Beijing 100083, China; 2 Liaoning Institute of Economic Forestry, Dalian 116031, China; 3 Institute of Forestry and Pomology, Beijing Academy of Agriculture and Forestry Science, Beijing 100097, China; 4 State Key Laboratory of Efficient Production of Forest Resources, Ministry of Education Key Laboratory of Silviculture and Conservation, Beijing 100083, China

**Keywords:** walnut, salicylic acid, cold tolerance, transcriptome, metabolome

## Abstract

Walnut (*Juglans regia*) is an economically significant woody oil tree species widely cultivated in China. However, its production is increasingly threatened by extreme low-temperature events, such as unseasonal frosts and late-spring cold. Salicylic acid (SA) is a key phytohormone known to enhance cold tolerance in plants, yet its underlying mechanism in walnut remains unclear. In this study, we present the first integrated analysis combining physiological measurements, transcriptomics, and metabolomics to investigate how exogenous SA improves cold tolerance in walnut leaves. Our results showed that SA treatment significantly increased the accumulation of soluble sugars, chlorophyll, and proline, enhanced peroxidase (POD) activity, and reduced malondialdehyde (MDA) levels under cold stress. Multi-omics analysis revealed that SA modulated the expression of genes involved in multiple hormone signaling pathways, including those of SA, auxin, jasmonic acid, and abscisic acid, and altered corresponding hormone levels. Notably, carbohydrate metabolism emerged as a central pathway mediating SA-induced cold adaptation. Weighted gene co-expression network analysis (WGCNA) further identified several core candidate genes, such as *JrTGA*, *JrPP2C*, *JrTPS*, and *JrBAM*, which may play key roles in this process. Collectively, this study provides the first multi-omics perspective on the regulatory network underlying SA-enhanced cold tolerance in walnut. These findings offer both a theoretical and technical foundation for applying SA in cold-resistant walnut cultivation and contribute to the development of stress-resilient production strategies.

## 1. Introduction

Walnut (*Juglans regia*) is the most widely cultivated economic tree species in temperate regions. As one of the world’s four major nuts, it holds considerable social, economic, and ecological importance [1]. China has the world’s largest walnut cultivation area, encompassing 8 million hectares, and leads global production with an annual output of nearly 6 million tons. As a strategically important woody oil species, walnut cultivation contributes significantly to rural income generation, poverty alleviation, and ecological sustainability [2]. Walnut kernels are rich in oil, protein, amino acids, and mineral elements, supporting their use in edible oil processing and functional food development [3,4]. Moreover, walnut exhibits considerable medicinal potential: various tissues—including the kernel, shell, and roots—contain high levels of bioactive compounds such as flavonoids, phenolic acids, melatonin, and juglone, underscoring its value as a pharmaceutical resource [5,6]. Despite its economic and nutritional importance, walnut production in China is increasingly constrained by late-spring frosts, which frequently damage new shoots, female flowers, and young fruits, leading to substantial yield losses or complete crop failure. Cold stress thus represents a major limiting factor for the sustainable and high-quality development of the walnut industry.

In the context of global climate change, extreme weather events have become increasingly frequent. The incidence and intensity of low-temperature stresses, such as abnormal winter cold and late-spring frost, continue to rise, establishing cold stress as a major abiotic factor limiting plant growth and development. Heightened temperature variability and the growing frequency of extreme freezing events pose significant threats to global plant production systems [7].

From a physiological perspective, cold stress impairs metabolic efficiency, disrupts normal growth and development, and induces excessive accumulation of reactive oxygen species (ROS). This oxidative burst can damage biomembranes and macromolecular structures, ultimately reducing plant productivity and, in severe cases, leading to plant death [8]. Walnut is particularly vulnerable to cold injury due to its large pith and high tissue moisture content. Sudden frost events can directly compromise flowering and fruit set, resulting in sharp declines in both yield and fruit quality, and thereby substantially diminishing crop economic value [4,9]. Therefore, elucidating the cold tolerance mechanisms in walnut is of considerable practical importance for developing strategies to enhance stress resilience and for ensuring the stable and sustainable development of the walnut industry.

Salicylic acid (SA) is a key endogenous hormone that not only regulates plant growth and development but also plays a central role in abiotic stress responses, functioning as an important modulator of stress tolerance [10]. Studies have confirmed that exogenous SA application can significantly improve the tolerance of various plants to abiotic stresses such as drought stress [11], high temperature stress [12], and salt stress [13]. These effects are primarily mediated through the induction of defense-related metabolites such as phenolic compounds, activation of the antioxidant system, and modulation of genes and enzymes involved in carbohydrate and energy metabolism [14,15].

To date, the regulatory role and underlying mechanism of exogenous SA in cold tolerance of walnut remain unclear. In particular, no integrated multi-omics study has elucidated the molecular pathways through which SA enhances cold resistance in this species. Previous research has been limited to physiological measurements, lacking a comprehensive investigation of the associated molecular networks. To address this gap, the present study employed an integrated approach combining physiological assays, transcriptomics, and metabolomics to investigate the responses of walnut leaves to cold stress following exogenous SA treatment. The objective was to identify key molecular pathways and core regulators involved in SA-mediated cold adaptation. The findings offer both practical guidance for SA application in cold-tolerant walnut cultivation and a theoretical basis for developing stress-resilient production systems. Moreover, this study advances the understanding of SA-regulated cold tolerance in woody perennials, providing new insights into the adaptive mechanisms of fruit trees under low-temperature stress.

## 2. Results

### 2.1. Effects of Exogenous SA on the Physiological Characteristics of Walnut Under Cold Stress

**Figure 1:**
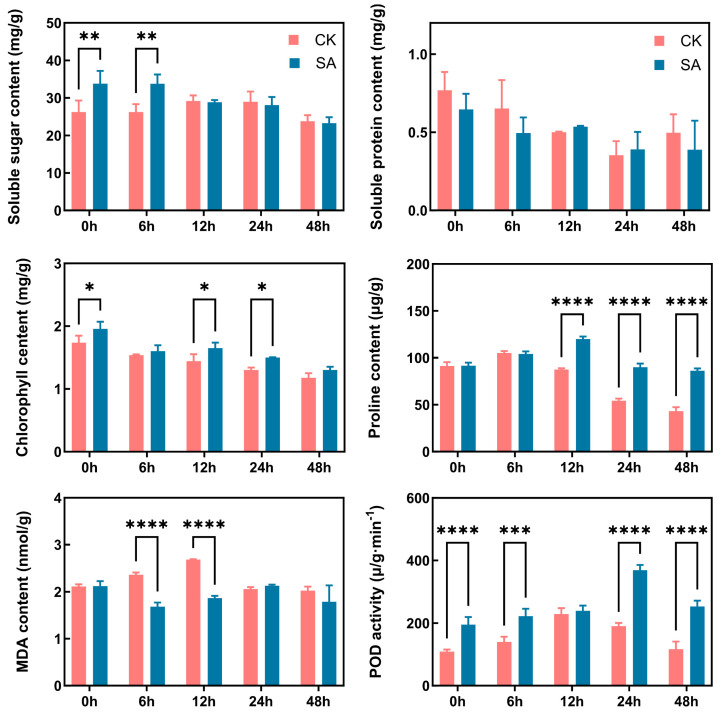
Effects of exogenous SA treatment on the physiological characteristics of walnut under cold stress. *, **, *** and **** represent significant or extreme difference at 0.05, 0.01, 0.001, and 0.0001 levels, respectively.

To assess the effect of exogenous SA on cold tolerance in walnut, we measured dynamic changes in soluble sugar, soluble protein, chlorophyll, MDA, and proline contents, as well as POD activity in leaves under cold stress (Figure 1). SA treatment significantly modulated the physiological responses of walnut seedlings to low temperature. Specifically, soluble sugar content was markedly higher in the SA-treated group than in the control during the early phase of cold stress; chlorophyll content became significantly elevated at the intermediate stage; and proline accumulation increased substantially from the middle to late stages. Although MDA levels were similar between groups before stress, SA treatment led to a significant reduction at 6 h and 12 h of cold exposure, indicating that SA alleviated membrane lipid peroxidation and reduced cold-induced membrane damage. In addition, POD activity was significantly higher in SA-treated plants at 0 h, 6 h, 24 h, and 48 h, suggesting that SA sustained an enhanced antioxidant capacity for reactive oxygen species (ROS) scavenging throughout the stress period.

### 2.2. Transcriptome Sequencing and Differentially Expressed Genes (DEGs) Analysis

To identify the cold-responsive genes in walnuts, transcriptome sequencing was performed on leaf samples collected at five time points (0, 6, 12, 24, and 48 h) under 4 °C cold stress. The results showed that each of the 30 samples yielded between 39,503,876 and 65,607,398 clean reads. The Q20 and Q30 values ranged from 98.87% to 99.18% and from 96.41% to 97.38%, respectively, confirming that the sequencing data were of high quality and suitable for downstream analysis (Table S1).

Clean reads from each sample were aligned to the reference genome, with mapping rates between 87.52% and 93.94%. In total, 29,194 expressed genes were identified, including 25,573 known and 3621 novel genes. Additionally, 57,251 expressed transcripts were detected, comprising 24,489 known and 32,762 new transcripts. Principal component analysis (PCA) further revealed clear clustering of biological replicates within each treatment group (Figure S1), indicating good experimental reproducibility and data reliability.

Analysis of differentially expressed genes (DEGs) revealed that in both the control (CK) and SA treatment groups, the number of DEGs gradually increased with prolonged cold stress, with a particularly marked rise in up-regulated genes (Figure S1). The SA treatment group exhibited a significantly higher number of up-regulated genes compared to the CK group, suggesting that SA treatment may enhance walnut leaf adaptation to cold stress through broader transcriptional activation. Examination of DEGs between the SA and CK groups at each time point revealed a dynamic pattern characterized by an initial decrease followed by a marked increase, peaking at 48 h of cold stress, at which point a total of 1078 DEGs were identified.

**Figure 2:**
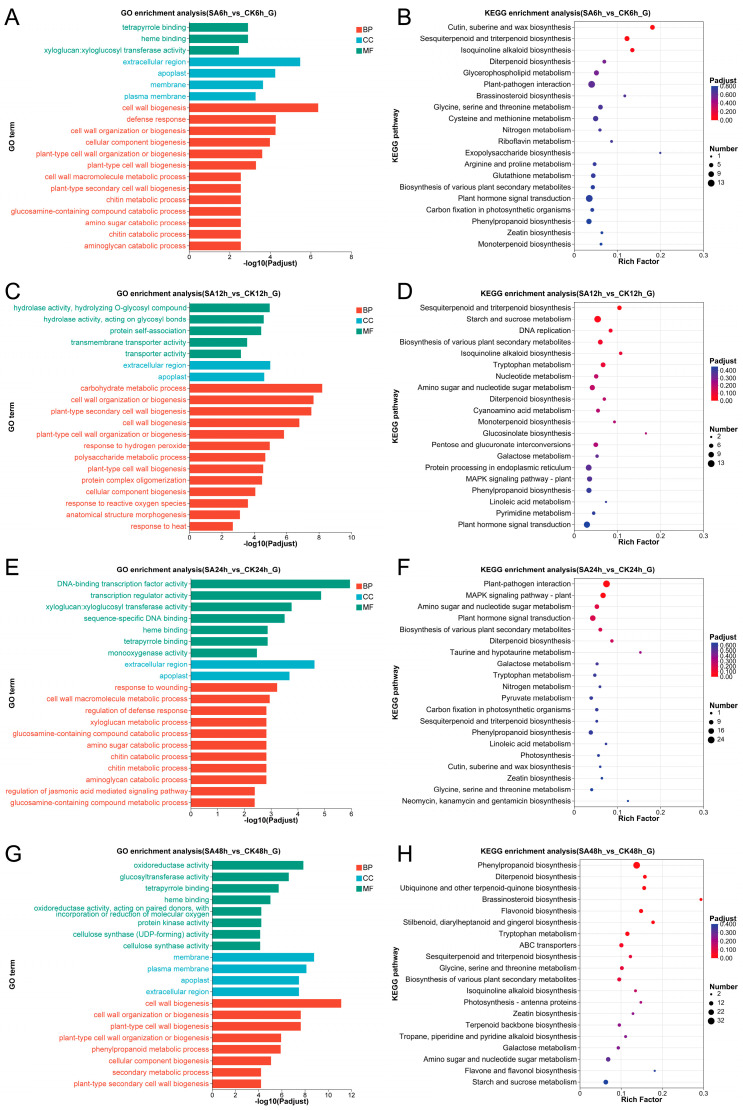
GO and KEGG enrichment analysis of DEGs for the comparison of the SA with the CK group. (**A**) GO enrichment analysis of SA6h vs. CK6h group; (**B**) KEGG enrichment analysis of SA6h vs. CK6h group; (**C**) GO enrichment analysis of SA12h vs. CK12h group; (**D**) KEGG enrichment analysis of SA12h vs. CK12h group; (**E**) GO enrichment analysis of SA24h vs. CK24h group; (**F**) KEGG enrichment analysis of SA24h vs. CK24h group; (**G**) GO enrichment analysis of SA48h vs. CK48h group; (**H**) KEGG enrichment analysis of SA48h vs. CK48h group.

GO functional enrichment analysis (Figure 2) showed that DEGs during each cold stress period were significantly enriched in biological process (BP) terms related to cell wall formation and decomposition, plant secondary cell wall biogenesis, and response to stress. At the molecular function (MF) level, DEGs were commonly enriched in categories such as hydrolase activity and transferase activity. Notably, at 24 h of cold stress, DEGs were specifically enriched in MF terms, including DNA-binding transcription factor activity and signal transduction activity. Moreover, DEGs identified between the SA and CK groups at 48 h showed significant enrichment in the oxidoreductase activity term. These results indicated that SA treatment may enhance cold tolerance in walnut leaves by regulating cell wall remodeling, stress-response pathways, key enzyme activities, and redox metabolism throughout the course of cold stress, thereby contributing to cellular homeostasis.

KEGG enrichment analysis exhibited time-dependent pathway enrichment patterns under cold stress (Figure 2). After 6 h, DEGs were primarily enriched in cutin, suberine and wax biosynthesis, sesquiterpenoid and triterpenoid biosynthesis, isoquinoline alkaloid biosynthesis, diterpenoid biosynthesis, and glycerophospholipid metabolism. At 12 h, enriched pathways included sesquiterpenoid and triterpenoid biosynthesis, starch and sucrose metabolism, DNA replication, Biosynthesis of various plant secondary metabolites, and isoquinoline alkaloid biosynthesis. By 24 h, DEGs were notably enriched in plant-pathogen interaction, MAPK signaling pathway—plant, amino sugar and nucleotide sugar metabolism, plant hormone signal transduction, and biosynthesis of various plant secondary metabolites. At 48 h, significant enrichment was observed in phenylpropanoid biosynthesis, diterpenoid biosynthesis, ubiquinone and other terpenoid-quinone biosynthesis, brassinosteroid biosynthesis, and flavonoid biosynthesis. Overall, the enriched pathways across time points consistently pertained to secondary metabolite synthesis (e.g., flavonoid biosynthesis and terpenoid biosynthesis), plant hormone signal transduction, and carbohydrate metabolism (e.g., starch and sucrose metabolism, and galactose metabolism). These findings indicated that SA treatment may enhance cold adaptation in walnut by modulating material and energy supply under cold stress, while concurrently strengthening stress defense through coordinated regulation of signaling networks and secondary metabolism.

### 2.3. Analysis of the Temporal Expression Trend of DEGs

**Figure 3:**
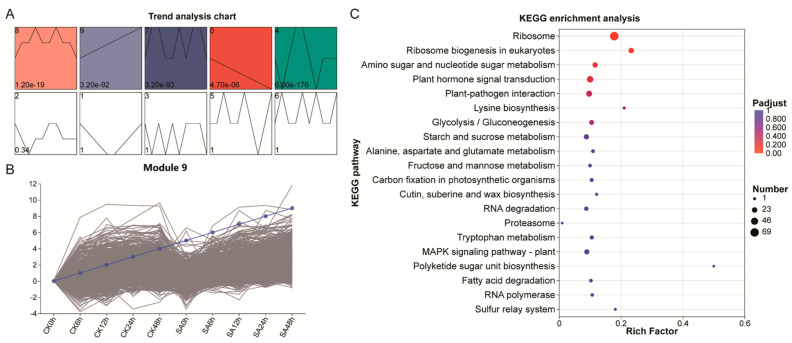
Time series expression of DEGs and functional enrichment. (**A**) Time-Series expression trends analysis of DEGs. (**B**) Gene expression trend map of module 9. (**C**) KEGG pathway enrichment analysis of genes in module 9.

Temporal expression profiling of DEGs identified 10 distinct temporal expression modules. Among these, modules 8, 9, 7, 0, and 4 exhibited significant expression trends (Figure 3A). Notably, the expression dynamics of the 1943 genes in module 9 displayed a sustained up-regulation pattern during cold stress in both CK and SA-treated groups (Figure 3B). KEGG enrichment analysis of this module revealed significant enrichment in the ribosome pathway, plant hormone signal transduction, and carbohydrate metabolism pathways (including glycolysis/gluconeogenesis, starch and sucrose metabolism) (Figure 3C). These results suggest that these pathways may function as core regulatory mechanisms underlying the SA-enhanced cold tolerance in walnut leaves.

### 2.4. Construction and Analysis of the Weighted Gene Co-Expression Network

**Figure 4:**
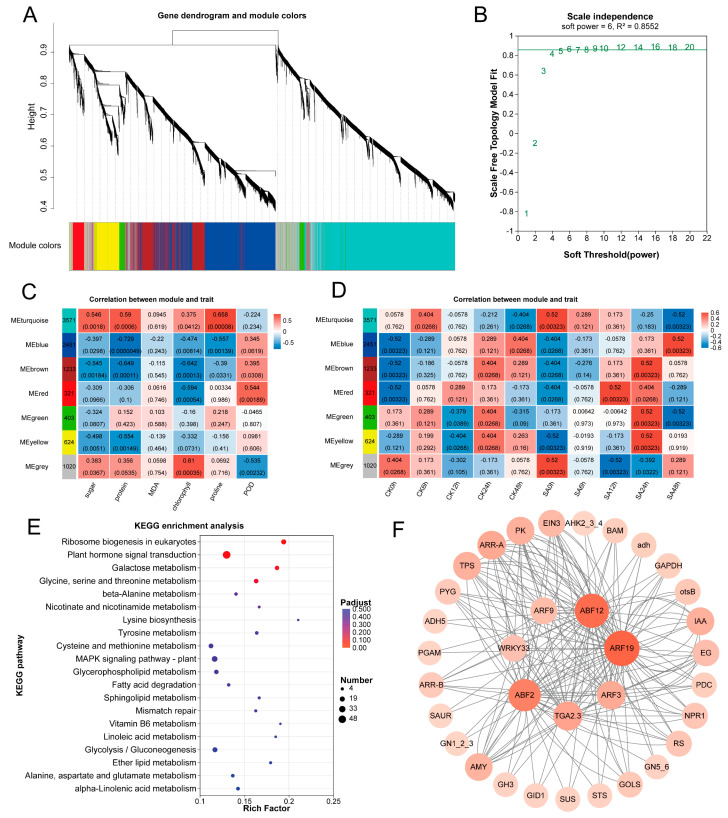
Weighted gene co-expression network analysis (WGCNA) of DEGs under cold stress. (**A**) Module clustering tree; (**B**) Scale independence; (**C**) Module–trait correlation analysis between co-expression network modules and physiological indicators; (**D**) Module–trait correlation analysis between the co-expression network module and cold stress duration; (**E**) KEGG enrichment analysis of genes in the MEblue module; (**F**) Co-expression network of the genes in the MEblue module, both node size and color intensity represent the degree value. Larger nodes and darker colors indicate higher degree values.

Based on RNA-Seq data and physiological response indicators obtained under cold stress, WGCNA was performed. The results clustered the DEGs into seven co-expression modules (Figure 4A). Among these, the MEturquoise, MEblue, and MEbrown modules exhibited significant correlations with cold-related physiological traits. Using the same analytical approach, correlation analysis was conducted with the cold stress duration as the phenotypic trait, revealing that the MEblue module showed the highest correlation with stress duration. Therefore, the MEblue module was selected as the core module for further investigation. KEGG enrichment analysis of genes in this module showed that DEGs were primarily enriched in plant hormone signal transduction, carbohydrate metabolism, and amino acid metabolism pathways (Figure 4E). Building on these findings, genes and transcription factors associated with plant hormone signaling and carbohydrate metabolism within this module were subjected to co-expression network analysis. The top 33 genes with the highest connectivity were identified as key candidate genes potentially involved in regulating cold tolerance in walnut (Figure 4F).

### 2.5. qRT-PCR Validation

**Figure 5:**
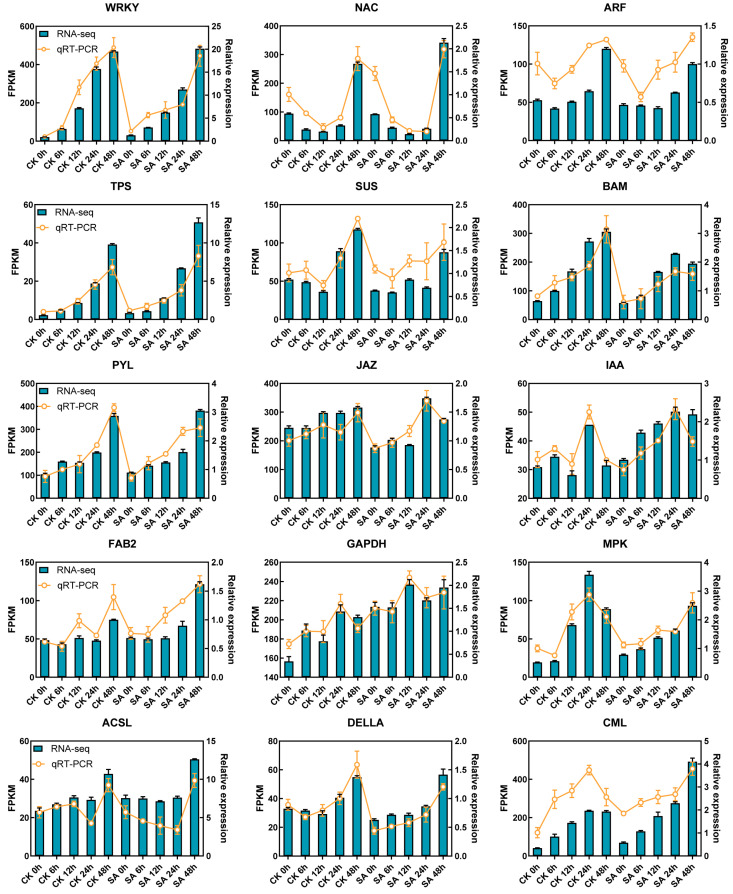
qRT-PCR validation of DEGs from RNA-seq data.

To validate the reliability of the transcriptome sequencing data, 15 DEGs were selected based on their involvement in the key pathways identified in this study for qRT-PCR analysis. The relative expression levels of these genes measured by qRT-PCR were highly consistent with the expression patterns obtained from RNA-seq (Figure 5). Furthermore, Pearson correlation analysis between the qRT-PCR and RNA-seq confirmed a strong positive correlation (Pearson correlation coefficient ≥ 0.793, *p*-value < 0.001; Table S2), demonstrating the accuracy and reproducibility of the transcriptomic data in this study.

### 2.6. Effects of Exogenous SA on the Metabolites of Walnut Under Cold Stress

**Figure 6:**
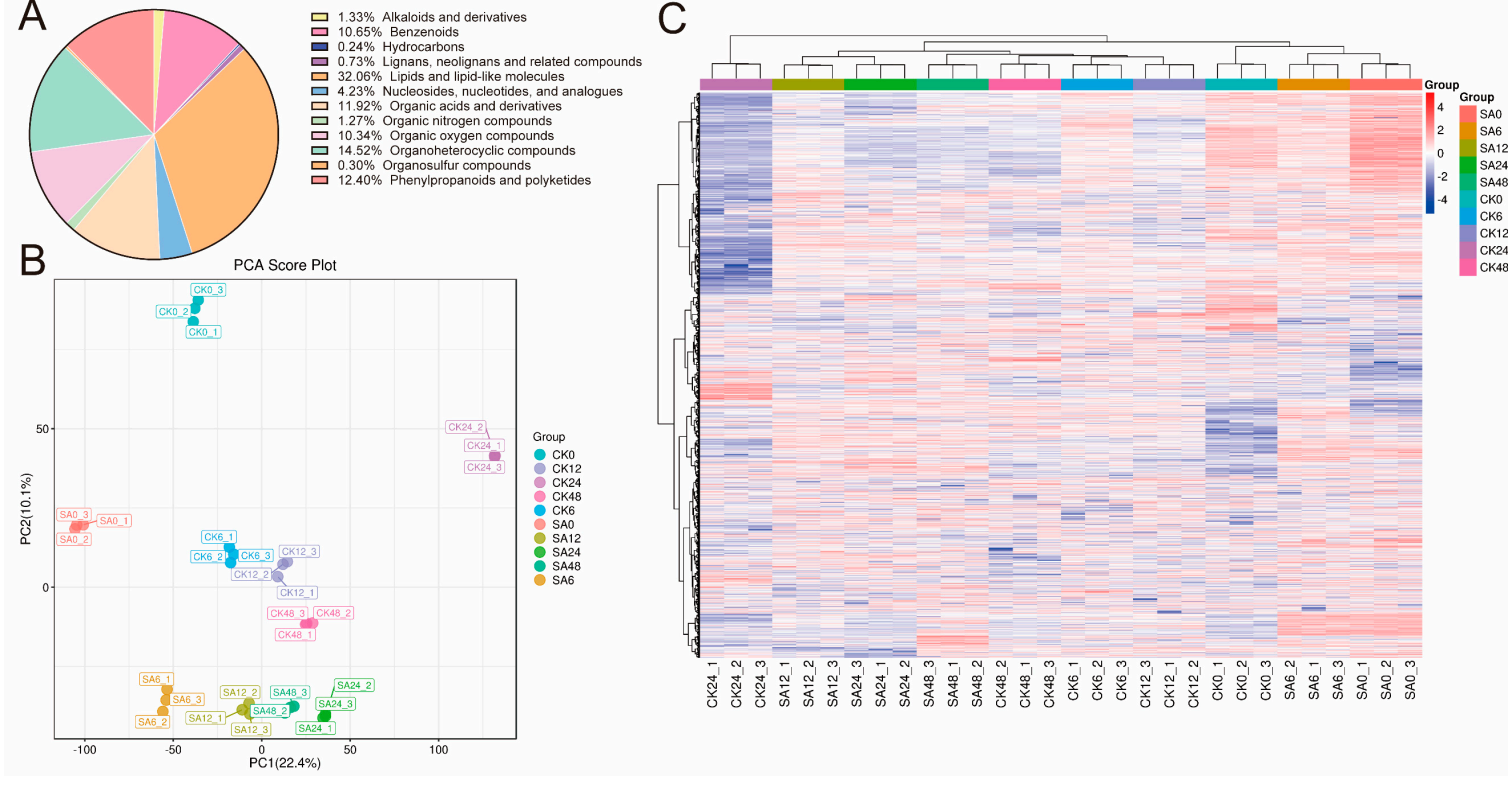
Metabolite mapping and sample analysis. (**A**) Pie chart of metabolite classification; (**B**) Principal component analysis of the sample; (**C**) Differential metabolite clustering heatmap.

A total of 1653 metabolites were detected across the 30 samples and classified into 18 categories (Figure 6A). Principal component analysis (PCA) showed clear separation between SA-treated and control groups in the principal component space, with tight clustering of biological replicates within each group, indicating high reproducibility of the metabolomic data (Figure 6B). This result also reflected significant differences in metabolite composition between the SA-treated and control groups. Furthermore, cluster heatmap analysis of metabolites (Figure 6C) showed distinct accumulation patterns between the SA and CK groups. It was consistent with the PCA results.

**Figure 7:**
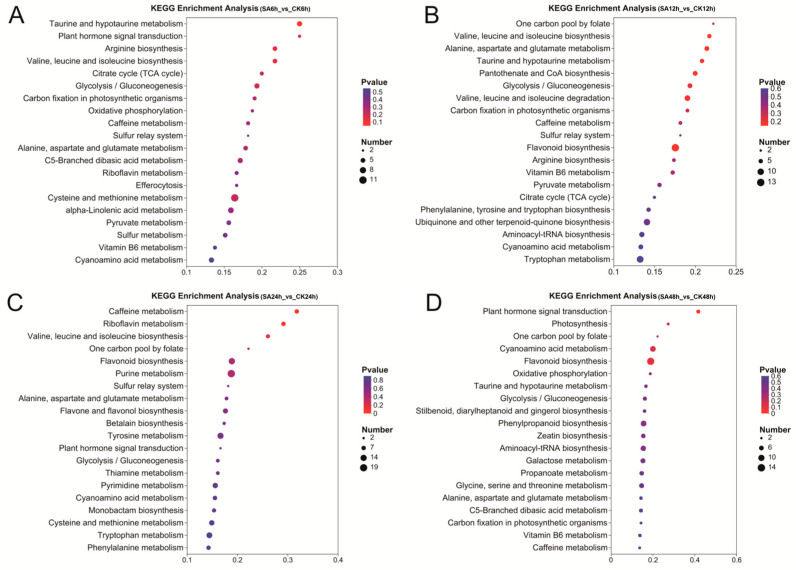
KEGG analysis of DAMs for the comparison of the SA with the CK group. (**A**) SA6h vs. CK6h group; (**B**) SA12h vs. CK12h group; (**C**) SA24h vs. CK24h group; (**D**) SA48h vs. CK48h group.

The KEGG enrichment analysis of differentially accumulated metabolites (DAMs) revealed time-dependent enrichment patterns across different stages of cold exposure. DAMs were predominantly enriched in pathways related to amino acid metabolism, plant hormone signal transduction, carbohydrate metabolism, and flavonoid biosynthesis (Figure 7). In summary, these findings indicate that SA treatment enhances cold tolerance in walnut leaves through the integrated regulation of material and energy homeostasis, stress-responsive signaling, and the synthesis of defensive secondary metabolites.

**Figure 8:**
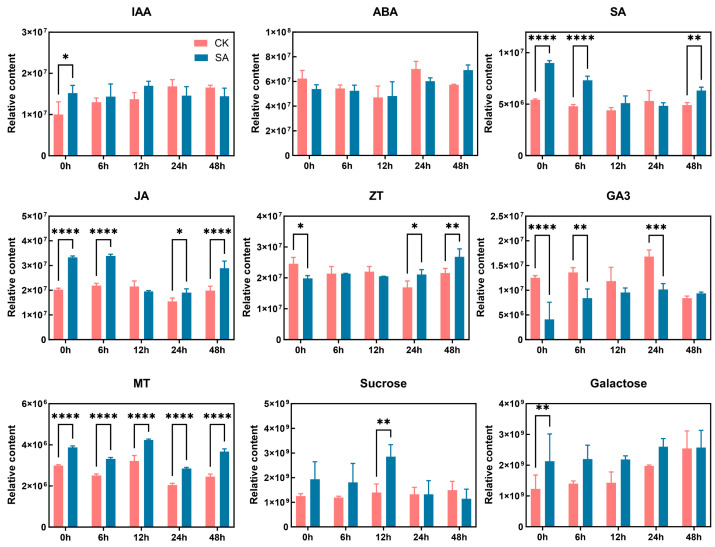
Effects of exogenous SA treatment on the hormone and sugar content of walnut leaves under cold stress. *, **, *** and **** represent significant or extreme difference at 0.05, 0.01, 0.001, and 0.0001 levels, respectively.

Overall, both DEGs and DEMs were primarily enriched in plant hormone signal transduction and carbohydrate metabolism pathways. To further investigate the response patterns within these core pathways, metabolites associated with these pathways were extracted from the metabolomics dataset and analyzed (Figure 8).

The results showed that SA treatment consistently promoted the accumulation of jasmonic acid (JA), melatonin (MT), and endogenous SA in walnut leaves under cold stress, while reducing gibberellin3 (GA_3_) levels. Indole-3-acetic acid (IAA) accumulation exhibited a transient increase followed by a decline, whereas zeatin (ZT) showed an opposite pattern, declining initially and then rising. Additionally, SA treatment enhanced sucrose and galactose accumulation during the early stress phase of cold stress. These findings suggest that SA enhances cold tolerance in walnut leaves through the synergistic and dynamic regulation of multiple hormones, together with early stress-stage carbohydrate mobilization that supports energy demands.

### 2.7. Analysis of Key Metabolic Pathways

**Figure 9:**
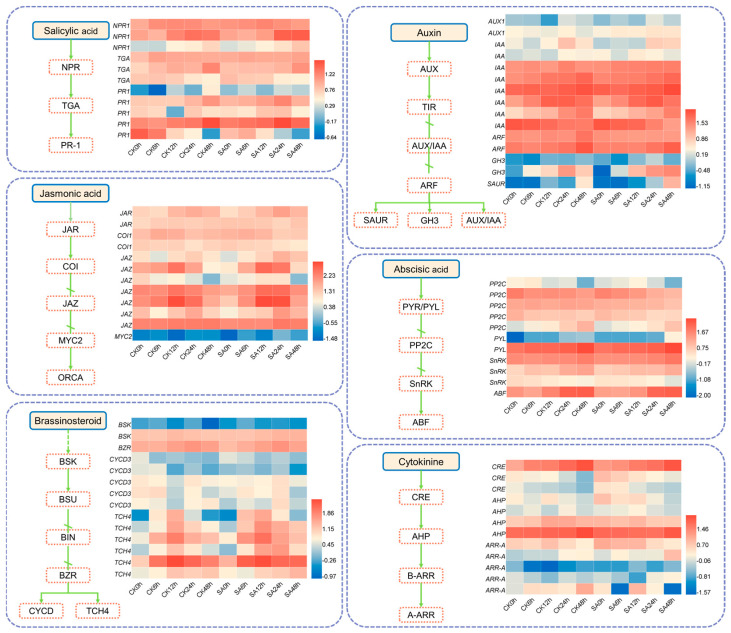
Plant hormone signal transduction pathway and gene expression patterns in walnut leaves under SA treatment and cold stress. → represents direct action; ⇢ represents indirect action; 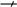 represents inhibition. Heatmaps show log10-transformed FPKM values of differentially expressed genes (DEGs).

Integrated transcriptomic and metabolomic analysis revealed that DEGs were significantly enriched in plant hormone signal transduction pathways (Figure 9, Table S3). Following SA treatment, hormone-related DEGs exhibited a pronounced up-regulation across multiple major hormone pathways. These included auxin-related genes (auxin/indole-3-acetic acid, *IAA*; auxin response factor, *ARF*; gretchen hagen 3, *GH3*; small auxin-up RNA, *SAUR*), a brassinosteroid-related gene (xyloglucosyl transferase TCH4, *TCH4*), JA-related genes (jasmonate resistant, *JAR*; jasmonate ZIM-domain, *JAZ*), abscisic acid-related genes (pyrabactin resistance-like, *PYL*; SNF1-related protein kinase, *SnRK*; ABA-responsive element binding factor, *ABF*), salicylic acid-related genes (nonexpressor of pathogenesis-related genes 1, *NPR1*; TGACG motif-binding factor, *TGA*; pathogenesis-related protein 1, *PR1*), and a cytokinin-related gene (Arabidopsis histidine phosphotransfer protein, *AHP*). In contrast, the abscisic acid (ABA)-related gene protein phosphatase 2C (*PP2C)* was down-regulated.

**Figure 10:**
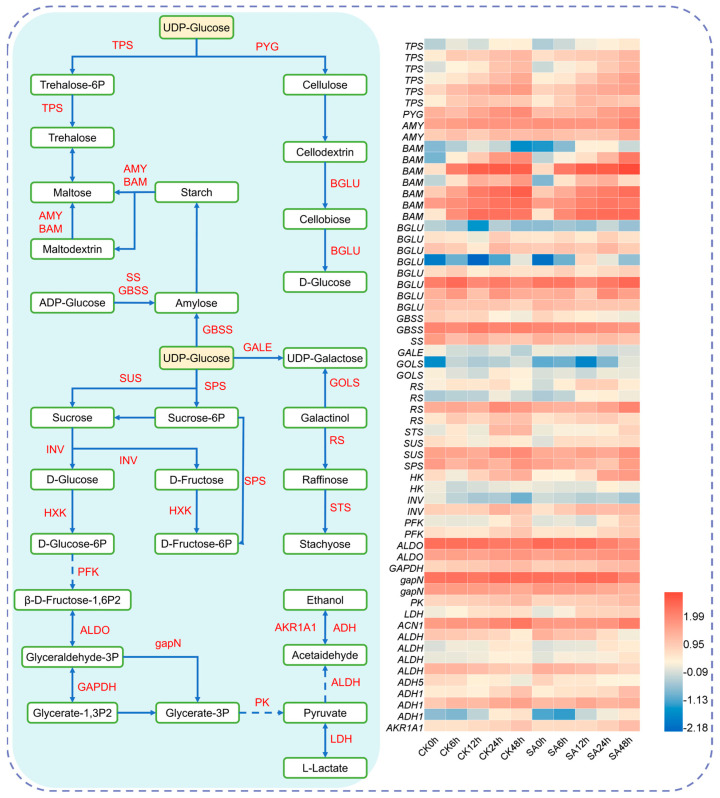
Carbohydrate metabolic pathway and gene expression patterns in walnut leaves under SA treatment and cold stress. → represents direct action; ⇢ represents indirect action. Heatmaps show log10-transformed FPKM values of differentially expressed genes (DEGs).

Under cold stress, plants require substantial energy to sustain normal growth and development. Carbohydrate metabolism serves as a primary source of low-molecular-weight soluble sugars under such conditions. In this study, integrated metabolomic and transcriptomic analyses revealed that both SA treatment and cold stress significantly influenced the expression of carbohydrate metabolism-related genes and the accumulation of associated metabolites, particularly in pathways such as starch and sucrose metabolism, galactose metabolism, and glycolysis/gluconeogenesis. (Figure 10, Table S4). Specifically, SA treatment up-regulated the expression of genes encoding amylase and sucrose synthase in walnut under cold stress, promoting the expression of genes involved in carbohydrate synthesis (sucrose synthase, *SUS*; sucrose phosphate synthase, *SPS*; trehalose-6-phosphate synthase, *TPS*; hexokinase, *HK*; starch synthase, *SS*; granule-bound starch synthase, *GBSS*) as well as genes related to carbohydrate breakdown (α-amylase, *AMY*; β-amylase, *BAM*; alkaline/neutral invertase, *INV*).

## 3. Discussion

Walnut is an economically important woody oil tree species, yet its production is increasingly constrained by low-temperature stress. In this study, we investigated the mechanisms underlying exogenous salicylic acid (SA)-enhanced cold tolerance using the widely cultivated walnut cultivar ‘Xiangling’, which is known to be susceptible to low-temperature stress and accounts for a significant proportion of production in major growing regions [16,17]. Through an integrated physiological, transcriptomic, and metabolomic approach, we demonstrate that SA treatment enhances cold tolerance in ‘Xiangling’ via coordinated regulation of hormone signaling and carbohydrate metabolism. These findings provide both mechanistic insights into SA-mediated cold adaptation and practical implications for cold-resistant walnut cultivation.

### 3.1. Analysis of Physiological Response Characteristics of Walnut Mediated by SA Under Cold Stress

SA functions as a key signaling molecule in plants, playing a central role in stress response networks and mediating abiotic stress tolerance [18]. In the present study, exogenous SA treatment significantly increased the accumulation of soluble sugars, chlorophyll, and free proline in walnut leaves under cold stress, enhanced POD activity, and effectively reduced MDA content. These physiological responses are consistent with findings from previous studies [19,20].

Soluble sugar accumulation is a well-documented protective mechanism in cold-adapted plants, serving functions as osmoregulators, cryoprotectants, and signaling molecules [21]. Elevated soluble sugar levels enhance stress tolerance by mitigating cold-induced damage, scavenging reactive oxygen species (ROS), and stabilizing membrane structures [22]. Previous research has further demonstrated that SA can positively regulate plant cold tolerance by modulating soluble sugar synthesis and accumulation [23].

In this study, exogenous SA treatment significantly promoted chlorophyll accumulation in walnut leaves under cold stress, suggesting that SA helps maintain photosynthetic capacity by enhancing chlorophyll biosynthesis. As a key osmolyte, proline preserves membrane integrity and cellular osmotic balance under low-temperature conditions, thereby mitigating cold-induced damage [24]. SA signaling promotes proline accumulation by activating the expression of proline biosynthesis genes such as delta1-pyrroline-5-carboxylate synthase (*P5CS*), underscoring its regulatory role in cold tolerance [25]. Abiotic stress damage is closely associated with oxidative injury at the cellular level. MDA, a product of membrane lipid peroxidation, serves as an indicator of membrane damage. POD, a key component of the antioxidant system, scavenges excess ROS and prevents oxidative stress under cold conditions [26]. In the present study, SA treatment significantly increased POD activity in walnut leaves during cold stress, indicating that SA alleviates oxidative damage, maintains membrane stability, and supports normal cellular metabolism.

### 3.2. Synergistic Regulation Mechanism of the SA-Mediated Multi-Hormone Signaling Pathway to Enhance Cold Tolerance

As key signaling molecules that regulate plant growth, development, and stress responses, plant hormones play a central role in mediating plant adaptation to cold stress [27]. This study found that exogenous SA treatment markedly altered the hormonal balance in walnut under cold stress: endogenous SA, JA, and MT levels significantly increased; IAA and ZT levels showed stage-specific up-regulation; GA3 content decreased notably, while ABA content remained largely unchanged. Correspondingly, the expression of marker genes in multiple hormone pathways was significantly induced, including *PR1* (SA pathway), *JAZ/MYC2* (JA pathway), *PYL/SnRK* (ABA pathway), and *B-ARR* (cytokinin pathway). These results indicated that exogenous SA treatment perhaps enhances cold tolerance in walnut through a coordinated regulatory network involving both hormone accumulation and signal gene expression.

In terms of the SA signaling pathway, exogenous SA treatment promoted the accumulation of endogenous SA, which facilitated the binding of *NPR1* to *TGA* transcription factors and activated the expression of the downstream defense gene *PR1*. This NPR1-TGA-PR1 signaling module contributed to enhanced cold tolerance in plants. The direct regulatory role of *NPR1* and *PR1* in plant responses to cold stress has been confirmed in previous studies [28,29].

As an upstream signaling molecule of the ICE-CBF (inducer of CBF expression- C-repeat binding factor) cascade, JA can positively regulate cold tolerance in *Arabidopsis thaliana* [30]. Within the JA signaling pathway, *JAZ* family genes modulate plant responses to biotic and abiotic stresses through complex regulatory mechanisms. Overexpression of *GsJAZ* in soybean has been shown to enhance alkaline and salt tolerance [31]. In the present study, SA treatment significantly increased JA content and generally up-regulated the expression of *JAZ* family members, suggesting a key mechanism underlying SA-enhanced cold tolerance in walnut.

Abscisic acid (ABA) is widely recognized as a central hormone regulating plant responses to cold stress. Within the ABA signaling pathway, the roles of key regulatory components have been well documented: *PP2C* functions as a negative regulator, while *PYL* and *SnRK* act as positive regulators [32]. In this study, the down-regulation of *PP2C* and up-regulation of *PYL* and *SnRK* further support the involvement of the ABA signaling pathway in walnut cold responses.

Under SA regulation, melatonin levels remained consistently elevated throughout the cold stress period. Melatonin has been shown to enhance antioxidant enzyme activity and up-regulate corresponding gene expression, thereby mitigating stress-induced damage [33]. It also improves cold tolerance in diverse species, including cucumber, tomato, watermelon, and *Arabidopsis* [34]. In contrast, gibberellin levels generally exhibit a negative correlation with plant cold tolerance [35].

Auxin content exhibited a stage-specific pattern, with an initial increase followed by a decline during later stages of cold stress, while most auxin pathway-related genes were up-regulated. Studies have shown that elevated endogenous IAA levels enhance cold tolerance in plants [36,37]. The up-regulated expression of key genes such as AUX/IAA and ARF in this study further supports this regulatory mechanism. Notably, IAA does not act alone in mediating stress responses but integrates stress resistance signals through crosstalk with hormones such as SA and melatonin, forming a multi-hormone synergistic network that collectively enhances cold tolerance in walnut [38].

### 3.3. The Mechanism of the SA-Mediated Carbohydrate Metabolism Pathway to Enhance Walnut Cold Tolerance

Carbohydrate metabolism functions not only as a central pathway for energy supply and material synthesis in plants but also as a key physiological network mediating cold stress responses. The results of this study showed that exogenous SA treatment significantly increased the contents of soluble sugars, sucrose, and galactose in walnut leaves under cold stress and altered the expression patterns of key genes in starch and sucrose metabolism pathways. Notably, the *BAM* was markedly up-regulated following cold stress, accelerating the conversion of starch to soluble sugars.

During cold adaptation, the rate of starch degradation is positively correlated with the accumulation of soluble sugars. These soluble sugars act as antioxidants to scavenge excess ROS induced by cold stress, and play a key role in maintaining cellular homeostasis, and enhance cold tolerance [39,40]. Among soluble sugars, sucrose functions both as an energy carrier and a stress-protective metabolite with potent antioxidant properties [41].

UDP-glucose is catalyzed by *TPS* to produce trehalose-6-phosphate and trehalose, both of which play important roles in plant growth, development, and stress adaptation. Trehalose, in particular, enhances plant tolerance to abiotic stress by protecting biomacromolecular structures and maintaining cellular integrity [42,43]. In this study, the expression of the *TPS* gene was significantly up-regulated under cold stress, consistent with previous reports [44]. This suggests that *TPS* may function as a conserved regulatory component in plant multi-stress responses. In walnut, *TPS* up-regulation likely promotes trehalose biosynthesis, thereby increasing soluble sugar levels, improving osmotic adjustment and antioxidant capacity, and ultimately enhancing cold tolerance.

Beta-glucosidase (*BGLU*) participates in multiple biological functions, including cell wall remodeling [45], responses to abiotic and biotic stresses [46,47], and the formation of lignin precursors [48]. Specifically, β-glucosidases, the enzymes encoded by *BGLU*, play a pivotal role in the metabolism of the plant cell wall. This observation aligns well with our GO enrichment results, which showed a significant enrichment in cell wall-related biological processes. It has been reported that *MsBGLU* was significantly up-regulated by low temperature [49], and a similar sustained up-regulation of *CaBGLU21* was observed under cold stress [50].

Raffinose family oligosaccharides (RFOs), including raffinose, play a significant role in plant responses to abiotic stress [51]. Plants accumulate substantial amounts of raffinose under cold stress, a process strongly associated with enhanced cold tolerance during cold acclimation [52]. Galactinol synthase (*GolS*), a key rate-limiting enzyme in RFO biosynthesis, regulates carbon partitioning between sucrose and RFOs [53]. *GolS* expression is markedly induced by cold stress in various species, including kidney bean seeds and *Arabidopsis thaliana* [52,54]. Furthermore, cold-induced high expression of raffinose synthase (*RS*) promotes raffinose accumulation in *Arabidopsis* leaves, thereby enhancing cold tolerance [55].

Integrating physiological, transcriptomic, and metabolomic data, this study demonstrates that exogenous SA enhances cold tolerance in walnut primarily through two interconnected pathways: plant hormone signal transduction and carbohydrate metabolism. In plant hormone signal transduction, SA treatment activated the NPR1-TGA-PR1 module of the SA pathway, the JAZ-mediated JA pathway, and the PYL/SnRK-regulated ABA pathway, while simultaneously modulating endogenous levels of JA and MT. This coordinated hormonal regulation strengthens stress signal perception and transduction. In carbohydrate metabolism, SA up-regulated key genes such as *TPS*, *BAM*, and *SUS*, accelerated starch breakdown, and promoted the accumulation of soluble sugars (e.g., sucrose), thereby improving energy supply and osmotic adjustment. Together, these responses enhance cellular antioxidant capacity, increase ROS scavenging efficiency, and reduce membrane lipid peroxidation. The two pathways functioned synergistically and formed an integrated regulatory network that systematically improved walnut adaptation to cold stress. However, the present study has certain limitations. Although integrated multi-omics analysis has identified key candidate genes, the interactions between key transcription factors and their target genes remain to be functionally characterized. Targeted experimental approaches are needed to validate these regulatory relationships and further elucidate the molecular mechanisms underlying the SA-mediated cold tolerance in walnut.

## 4. Materials and Methods

### 4.1. Plant Materials and Experimental Design

The experiment was conducted in a walnut orchard at the experimental base of the Liaoning Economic Forest Research Institute. Two-year-old grafted seedlings of the ‘Xiangling’ walnut cultivar were used as plant materials. To ensure experimental validity and reproducibility, healthy seedlings with uniform growth and no signs of pests or diseases were selected. Seedlings were cultivated at 25 °C with 70% relative humidity under a 16/8 h light/dark photoperiod and a light intensity of 80 µmol·m^−2^·s^−1^. Walnut seedlings in the treatment group were sprayed with a 200 mg/L SA solution, while those in the control group received an equal volume of water. This concentration was selected based on previous studies in woody plants demonstrating that 200 mg/L of SA effectively enhances stress tolerance without causing phytotoxicity [56]. Spraying was performed once every two days for a total of three applications, with a volume of 1 L per treatment group. Each treatment included ten seedlings as biological replicates. Following treatment, all plants were subjected to low-temperature stress at a constant 4 °C [57]. Leaf samples were collected at designated time points (0, 6, 12, 24, and 48 h) after stress initiation for subsequent experimental analyses. The 0 h samples, collected immediately before cold stress exposure, served as the non-stressed control and reflected the effects of SA treatment alone under normal growth conditions (25 °C).

### 4.2. Physiological Index Determination

The chlorophyll content was measured using ethanol extraction colorimetry [58].The soluble sugar content was determined using the anthrone reagent method [59]. Soluble protein content was determined by the Coomassie Brilliant Blue G- 250 method [60]. The thiobarbituric acid method was used to determine the malondialdehyde (MDA) content [61]. Free proline was measured according to the method by Bates et al. [62]. Peroxidase (POD) activity was estimated using the guaiacol oxidation colorimetric method [63]. All measurements were performed using a UV-2600 spectrophotometer (Shimadzu, Kyoto, Japan), and the determination was performed in triplicate for each sample.

### 4.3. Statistical Analysis

The data was analyzed using single-factor ANOVA and the minimum significance difference (LSD) method for multiple comparisons. A *p*-value of less than 0.05 was used as the threshold for determining significant differences.

### 4.4. RNA Extraction and Sequencing

Total RNA was extracted from walnut leaves using the Omega Bio-Tek Plant RNA Kit (Omega Bio-Tek, Norcross, GA, USA). RNA concentration and purity were determined using a NanoDrop 2000 spectrophotometer (Thermo Fisher Scientific, Waltham, MA, USA), and RNA integrity was assessed using the Bioanalyzer 2100 system (Agilent Technologies, Santa Clara, CA, USA). mRNA was purified from total RNA using Oligo (dT) magnetic beads and fragmented for library construction. Following library preparation, fragments were enriched by PCR amplification. Library quality was verified using the Agilent 2100 Bioanalyzer (Agilent Technologies, Santa Clara, CA, USA), and library concentration was quantified by fluorescence detection to ensure quality control. After RNA extraction, purification, and library construction, these libraries were sequenced using next-generation sequencing (NGS) based on the Illumina sequencing platform.

### 4.5. Quality Control and Identification of DEGs

FASTp was used to filter raw reads and remove low-quality sequences, generating high-quality clean data. The clean data were aligned to the walnut reference genome (http://aegilops.wheat.ucdavis.edu/Walnut/annotation/ (accessed on 1 June 2025)), and the read distribution on the genome was statistically compared. Gene and transcript expression levels were quantified using RSEM. Differential expression analysis was performed with DESeq, and genes with |log_2_Fold Change| > 2 and an adjusted *p*-value < 0.05 were considered differentially expressed genes (DEGs). Functional annotation and pathway enrichment analyses were conducted using the Gene Ontology (GO) and Kyoto Encyclopedia of Genes and Genomes (KEGG) databases.

### 4.6. STEM Time-Series Trend Analysis

Short Time-Series Expression Miner (STEM) analysis was performed on the transcriptome data collected at multiple time points (0, 6, 12, 24, and 48 h) under cold stress to identify statistically significant temporal expression patterns [64]. This analysis enabled the profiling of dynamic expression trends of DEGs across the sampling time points. Genes with similar expression patterns were subsequently clustered using the k-means algorithm to identify distinct temporal expression modules associated with cold stress response.

### 4.7. Weighted Co-Expression Network Analysis (WGCNA)

WGCNA was performed to explore regulatory interactions among DEGs and to identify key candidate genes [65]. RNA-Seq gene expression data (FPKM > 1) from the 30 leaf samples were used to construct a scale-free co-expression network. Physiological data were incorporated as phenotypic traits for module–trait association analysis. The soft-thresholding power was set to 6. The resulting co-expression network was visualized using Cytoscape (version 3.7.1).

### 4.8. Quantitative Real-Time PCR Analysis (qRT-PCR)

To validate the reliability of transcriptome sequencing data, 15 DEGs were selected for qRT-PCR analysis. qRT-PCR was performed on an ABI 7500 real-time PCR system (Applied Biosystems, Foster City, CA, USA) using 18S rRNA as an internal reference gene [66]. Primers were designed using Primer 6.0 software (Table S5). The reaction mixture was prepared according to the Super Real Pre Mix Plus (SYBR Green) kit instructions (Tiangen, Beijing, China). Three technical replicates were included for each gene to ensure data stability and reproducibility. Relative gene expression was calculated using the 2^−∆∆Ct^ method [65]. To assess the consistency between qRT-PCR and RNA-seq data, Pearson correlation analysis was performed using SPSS software, version 28. The Pearson correlation coefficient and *p*-value were calculated to evaluate the strength and significance of the correlation.

### 4.9. Untargeted Metabolomic Profiling

Leaf samples were ground to a fine powder in liquid nitrogen and homogenized in 70% methanol. The homogenate was centrifuged at 20,000 rpm for 20 min at 4 °C, and the supernatant was filtered through a 0.22 μm nylon syringe filter. Liquid chromatography (LC) analysis was performed using a Vanquish UHPLC System (Thermo Fisher Scientific, USA), and mass spectrometric detection was carried out on an Orbitrap Exploris 120 (Thermo Fisher Scientific, USA) equipped with an electrospray ionization (ESI) source. Pooled quality control (QC) samples were prepared by mixing equal aliquots of all samples and injected at regular intervals throughout the analytical run to monitor instrument stability and signal drift. Metabolites detected in QC samples with a relative standard deviation (RSD) > 30% were excluded from further analysis. All metabolite levels are reported as relative peak areas normalized to internal standards and QC samples, which is appropriate for comparative analysis in untargeted metabolomics. Metabolites were identified by accurate mass and MS/MS spectral matching against public databases, including HMDB (http://www.hmdb.ca (accessed on 7 June 2025)) [67], massbank (http://www.massbank.jp/ (accessed on 7 June 2025)) [68], KEGG (https://www.genome.jp/kegg/ (accessed on 7 June 2025)) [69], LipidMaps (http://www.lipidmaps.org (accessed on 7 June 2025)) [70], and mzcloud (https://www.mzcloud.org (accessed on 7 June 2025)) [71]. Multivariate analyses, including principal component analysis (PCA), partial least squares-discriminant analysis (PLS-DA), and orthogonal PLS-DA (OPLS-DA), were performed using the ropls package in R. Model validity was assessed through permutation testing. Differentially accumulated metabolites (DAMs) were identified based on three criteria: variable importance in projection (VIP) score ≥ 1.0 from the OPLS-DA model, a *p*-value ≤ 0.05 from Student’s *t*-test, and fold change (FC) ≥ 2 [72]. Metabolites satisfying all conditions were considered DAMs. KEGG pathway enrichment analysis for DAMs was subsequently performed based on the hypergeometric distribution test.

## 5. Conclusions

This study provides a comprehensive understanding of the physiological and molecular mechanisms by which exogenous salicylic acid (SA) enhances cold tolerance in walnut, using an integrated approach combining physiological assays, transcriptomics, and metabolomics. SA treatment significantly increased the accumulation of soluble sugars, chlorophyll, and proline, enhanced POD activity, and reduced MDA levels under cold stress. Integrated multi-omics analysis further revealed that DEGs and DAMs were predominantly enriched in plant hormone signal transduction and carbohydrate metabolism pathways. Key candidate genes potentially involved in SA-mediated cold tolerance were also identified. These findings offer both a theoretical and practical foundation for the application of SA in cold-resistant walnut cultivation and contribute to a broader understanding of stress response regulation, supporting the development of stress-resilient production practices in walnut.

## Data Availability

The data presented in this study are available from the corresponding author upon reasonable request.

## Supplementary Materials

The following supporting information can be downloaded at: https://www.mdpi.com/article/10.3390/ijms27072948/s1.
