# Supplementary figures and images for: Comparative Transcriptomics and Metabolomics Analysis Revealed the Mechanism of Exogenous Salicylic Acid Improving the Cold Tolerance of Walnut
Source: Int J Mol Sci. 2026 Mar 24;27(7):2948. doi: 10.3390/ijms27072948 (PMC13072996; doi:10.3390/ijms27072948)

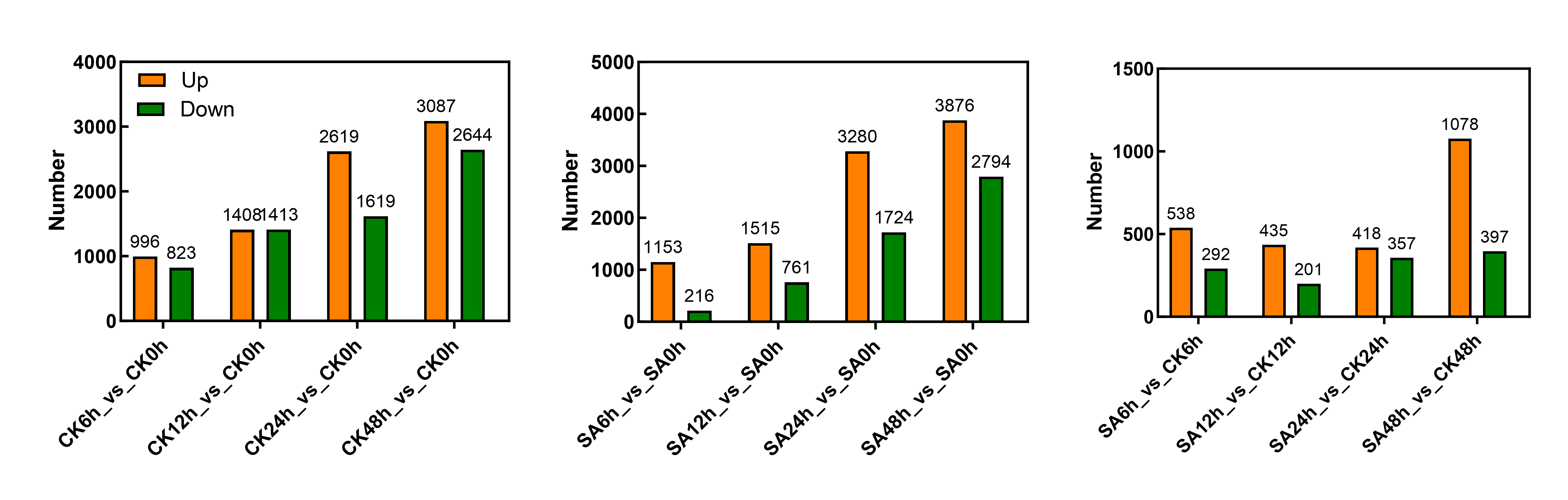

Supplement: Supplementary file 1 [file ijms-27-02948-s001.zip › Figure S1 Statistics of differentially expressed genes (DEGs) between the CK group and SA group at different stages of cold stress.tif]
